# Cepharanthine action in preventing obesity and hyperlipidemia in rats on a high-fat high sucrose diet

**DOI:** 10.1016/j.jsps.2022.09.013

**Published:** 2022-09-21

**Authors:** Adnan Iqbal, Rahila Najam, Shabana Simjee, Azfar Athar Ishaqui, Salman Ashfaq Ahmad, Zeeshan Ahmed, Shayan Ahmed, Salman Ahmed, Lailoona Jaweed, Madiha Maboos, Mir Muhammad Uzairullah, Suleha Jabeen, Muhammad Imran

**Affiliations:** a Department of Pharmacology, University of Karachi, Karachi, Pakistan; b HEJ Research Institute of Chemistry, Karachi, Pakistan; c Department of Pharmacy, Iqra University, Karachi, Pakistan; d Department of Pharmaceutics, University of Sindh, Jamshoro, Pakistan; e Department of Pharmaceutics, Jinnah University for Women, Karachi, Pakistan; f Department of Pharmacognosy, University of Karachi, Karachi, Pakistan

**Keywords:** Cepharanthine, ABCC10, Hyperlipidemia, Gene expression, Obesity

## Abstract

**Background:**

It was demonstrated that cepharanthine (CEP), derived from Stephania cepharantha hayata, is a potent inhibitor of the ABCC10 transmembrane protein. It is approved to be a natural product or remedy. The present study focuses on investigating whether cepharanthine effectively reduces hyperlipidemia and obesity in an experimental hyperlipidemic rat model.

**Method:**

Four groups of Wistar rats were assigned randomly to the following groups: a high-fat high sucrose diet (HFHS), normal-fat diet (NFD), HFHS plus cepraranthine (10 mg/kg) (HFHS-C), and a HFHS diet with atorvastatin (HFHS-A). The responses of rats were observed on the basis of serum and hepatic biochemical parameters, food intake, and body weight after CEP treatment, and assessing the histopathological modifications by the optical microscope in the liver and its cells.

**Results:**

Significant improvement in the serum total cholesterol (TC), serum triglycerides (TG), and serum low-density lipoprotein (LDL) levels were observed following CEP treatment. We have also observed significant improvement in the structure of liver tissue and reduced-fat droplets in the cytoplasm. Moreover, CEP had a significant effect in preventing the gain in body weight of animals, and food intake was not significantly affected.

**Conclusion:**

Our research results revealed that CEP significantly improved dyslipidemia and prevented the accumulation of fatty deposits in the rats' liver tissue fed an HFHS diet. In addition, CEP exerted an anti-obesity effect.

## 1 Introduction

There has been a sharp and steady increase in obesity in the past several years, making it a public health issue globally. Over 650 million people out of 1.9 billion are obese worldwide. The cause of obesity is an imbalance between energy expenditure and caloric intake. The worldwide obesity epidemic is due to several factors, mainly due to lifestyle changes, which have led to an increase in dietary fats consumption and a reduction in physical activity(Flier, 2004). The obesity epidemic leads to an aging population and lower life expectancy, as well as an increase in cancer, diabetes, hypertension, and imbalanced hormones in women that results in dyslipidemia and sterility, all of which are connected to the elevated risk of myocardial infarction and subsequent heart failure(American Heart, 2002). Various metabolic disorders, such as insulin resistance, are associated with obesity. Several factors contribute to the etiology of this disease, such as energy-rich diet consumption, psychological factors, lack of exercise, and sedentary lifestyles(Rippe et al., 1998). Obesity is challenging to treat due to its etiology, which remains obscure. The problem is further complicated by the unavailability of drugs suitable for treating this disorder and the limited side effects and short-term efficacy of the existing drugs(SURENDRAN et al., 2020).

Hyperlipidemia refers to abnormally high levels of lipids and lipoproteins in the blood. Several diseases are strongly associated with it, including coronary heart disease, arteriosclerosis, chronic kidney disease, myocardial infarction, and stroke. (Farnier and Davignon, 1998, Jain et al., 2007). In recent decades, cardiovascular disease (CVD) has become one of the leading causes of early death and illness in patients with dyslipidemia (Mitchell et al., 2016, Nelson, 2013). The need for new therapeutic options to prevent and manage dyslipidemia to reduce atherosclerosis and cardio-cerebral vascular disease is therefore imperative.

The most effective treatments for hyperlipidemia are medication, diet modification, smoking cessation, and exercise. In reducing lipids and lipoproteins in the blood, bile acid sequestrants, nicotine and statins are the most frequently prescribed medications. These drugs have proven beneficial in patients trying to prevent cardiovascular diseases (Zambon et al., 2014). Despite their effectiveness in modulating hyperlipidemia in clinical and preclinical studies, their adverse effects cannot be ignored, which occur on the kidney and liver, motivating scholars and researchers to develop further beneficial medicines to be used in the treatment of hyperlipidemia. (An and Yang, 2006, Wu et al., 2007, Xu et al., 2014). Many scholars have been attracted to Chinese medicine in recent years because of its limited side effects and stabilizing effect (An and Yang, 2006, Wu et al., 2007).

In contrast to other drugs, Cepharanthine (CEP), derived from Stephania cepharantha hayata, which is known to be a biscoclaurine alkaloid, is demonstrated to be ABCC10 transmembrane protein’s potent inhibitor(Zhou et al., 2009). The drug is approved to be a natural product or remedy. It has been used in Japan for over 70 years to treat chronic and acute diseases such as malaria, alopecia, venomous snakebites, and other afflictions with minimal side effects. Different biological effects are attributed to CEP, which is responsible for the clinical manifestations and activities. Stephania cephalantha plant is native to both Taiwan and China, where it has been utilized as Chinese herbal medicine or as an indigenous medicine. Initially, the use of the plant was reported by Bunzo Hayata in 1914, who was a botanist at the Taipei Imperial University (Bailly, 2019). The underlying mechanisms of CEP may contribute to the halting of hyperlipidemia and obesity and their prevention, but they have yet to be determined. As a result, the present study examines the protective role of CEP in hyperlipidemia and obesity in rats. Cepharanthine has never been studied for its lipid-lowering and anti-obesity properties.

## 2 Material and methods

### 2.1 Selection of experimental animals

A group of male Wistar albino rats weighing 150–175 g was obtained from the Dow medical college Ojha campus animal house in Karachi for experimental purposes. Acclimatization of the animals was performed at a temperature of 25 ± 2 °C with a relative humidity of (50 ± 15 %) after the purchase of the animals. Under the standard laboratory conditions (12 h light and dark each: night and day cycle), we kept the rats in polypropylene cages where every cage consisted of 6 rats. The animals were also given a normal diet and ad libitum access to tap water. In addition, these animals were handled per the protocol mentioned by the National research council (NRC) (Council, 1997). The Board of Advanced Studies and Research (A.S.R.B) of the University of Karachi approved the research proposal {ASRB/No. /04681/ Pharm.).

### 2.2 Drugs and experimental design

Rats were fed a diet high in fat and sucrose composed of three ml Banaspati ghee and one ml coconut oil given in a dose of 10 ml/kg, and 25 % sucrose was added to their drinking water bottles. Normal Fat Diet was fed to control rats (NFD Diet), which consisted of 70% carbohydrate, 20% protein, and 10% fat (Nissankara Rao et al., 2021). NFD diet is made up of crude protein (20%), crude oil (10%), Ash (10%), sand silica (0.15%), and crude fiber (4%) (Solanki & Bhatt, 2010). We used analytical-grade chemicals for all other chemical reactions. To test the biochemical reactions, we used distilled water in all cases.

The acclimatization of all the animals was done. They were further distributed into four different groups where each group consisted of 6 animals, and they were subjected to the following treatment: There were four groups: Group I were normal healthy controls consuming normal fat diets (NFD diets); Group II ate high-fat high sucrose diets (HFHS diets); Group III ate high-fat high sucrose diets, and Cepharanthine 10 mg/kg intraperitoneally started from 8th week for 60 days (HFHS + Cepharanthine); Group IV ate HFHS plus Atorvastatin 10 mg/kg orally as a standard drug from the 8th week for 60 days. A previous report determined the dose of cepharanthine and atorvastatin (Gosain et al., 2010, Samra et al., 2016). Rats were weighed weekly to monitor the changes in body weight, and the sample of blood from the rat tail was taken at different intervals to measure changes in plasma lipids. We also measured and calculated daily food intake to determine any differences in energy intake.

### 2.3 Estimation of lipid profile and liver enzymes in serum

A commercially available enzyme kit (HUMAN, Germany) was used to determine the serum's AST (aspartate aminotransferase), ALT (alanine aminotransferase), HDL, LDL-C, TC, and TG. The fraction of LDL-C, as well as the atherogenic index (AI), were determined depending as proposed by Friedewald (Friedewald, Levy, & Fredrickson, 1972)(Friedewald et al., 1972):

AI=LDL-C/HDL-C

LDL-C=TC/HDL-C

### 2.4 Histopathological analysis

A ketamine overdose (75 mg/kg) was administered to the rats when the experiment ended (Molina et al., 2015). A cardiac puncture was used to collect blood, and a surgical procedure was used to remove the livers. In this study, fat depots of the gonadal and the liver were dissected, and the masses were articulated as total mass (g) as well as fractional mass (g/100 g) (relative to total body mass). The Haug & Hostmark method was used for analyzing the hepatic lipids in liver samples (Haug & Høstmark, 1987). The hepatic tissues of each rat were homogenized in isopropanol at a concentration of 5 % (w/v). For extracting cholesterol and triglycerides, the homogenates were kept for 2 days at 4 °C; it was further centrifuged at 3000 revolutions per minute for 10 min.

Moreover, the cholesterol and Hepatic triglycerides were determined in the supernatant aliquots using the same commercial kits previously used to analyze the serum lipid. Slices of the liver were fixed by buffered formalin 10%, embedded in paraffin wax, and the liver samples were examined histopathologically by hematoxylin and Eosin staining (H & E). The histopathological slides were examined under a microscope (Olympus Optical, Tokyo, Japan).

### 2.5 Blood sugar level after fasting (FBG)

After overnight fasting, glucose levels in the blood were measured using a glucometer (Accu-chek, Roche, Switzerland). By nicking a rat’s tail tip, blood glucose levels were measured as mg/dL (Augustine et al., 2014).

### 2.6 Fasting serum insulin

Crystal ChemInc (Illinois, USA) provided an ultrasensitive rat insulin ELISA kit to measure serum insulin. The detection range is between 0.1 and 64 ng/ml. Insulin antibody exhibits 100 % cross-reactivity with rat insulin. In nanograms per milliliter, the results were reported. (Narasimhan et al., 2015).

### 2.7 Insulin resistance (HOMA-IR)

It was determined by multiplying (serum insulin in a fasted state and blood glucose fasting) by /405.(Matthews et al., 1985).

### 2.8 Anti-oxidant activity of Liver Malondialdehyde (MDA) and Superoxide Dismutase (SOD)

A small section of the liver was homogenized in cold phosphate buffer saline. After centrifugation of the homogenized solution, the supernatant was collected to determine the activity of MDA by thiobarbituric acid (TBA) and SOD activity by the xanthine oxidase method. The kit was purchased from Nanjing Jiancheng Bioengineering Institute, Nanjing, China, and we followed the instructions provided in the manual.

### 2.9 ABCC10 expression by Quantitative Real-Time-Polymerase Chain Reaction (qRT-PCR)

A total RNA extraction kit obtained from Thermo Fisher Scientific, USA was used to extract the total RNA from 50 mg of frozen liver tissues. A Nanodrop spectrophotometer (Thermo Fisher Scientific, USA) was used to measure the quality and quantity of RNA. QuantiTect reverse transcription kit (Two-Step RT-PCR Kit, Thermo Fisher Scientific, USA) was used to convert two mg of total RNA into cDNA. QuantiTect SYBR Green I PCR (Thermo Fisher Scientific, USA) was used for RT-PCR. Primer Blast was used in designing the primer of ABCC10, whose forward primer sequence is (5‘CTAGAACAGTGCCACCTGAGT-3‘), and the reverse is (5‘GTCTGTCTTCTGGTCCACACT-3‘). The sequence of Forward primer for GAPDH is (5‘-AGCGCAGAACATCATCGCTG-3‘), and the reverse is (5‘-CACCACGTCCTTGATGTCATC-3‘). Synbio Tech, NJ, 08852, USA, provided the primers. In the qRT-PCR program, the denaturation time was 15 s at 94C, the annealing time was 30 s at 55C, and the extension time was 30 s at 72C. Target gene copy number is stated as relative copy number (RCN) when standardized to GAPDH Ct value. A relative amount of gene amplification product was estimated using Livak KJ, Schmittgen TD equation 2DDCT.

### 2.10 Statistical analysis

Means ± standard deviation (SD) were used to represent the data. We conducted one-way analyses of variance (ANOVA) using SPSS 21.0 for statistics. Tukey's multiple comparison test was used to determine whether there were differences between the groups. Data were considered significant when the P-value was < 0.05.

## 3 Results

### 3.1 Cepharanthine prevented diet-induced obesity in rats fed a high-fat high sucrose diet

To investigate the Cepharanthine effect on obesity, the examination of the gonadal and perirenal fat mass index, food intake, and weight gain of the body was done for rats who were maintained on an HFHS diet. No significant differences were determined in body weights among the four groups at the initial stage of the experiment (Table 1).

**Table 1:** Influence of HFHS-C on the intake of food and weight of rats (n = 6).

Group	Initial wt (g)	4th week	8th week	12th week	16th week	Weight gain during Treatment(g)	Food Intake(g/d)
NFD	162.6 ± 8.3	190.6 ± 6.8	203.6 ± 13.8	221.8 ± 22.5^**^	239.8 ± 22.5^**^	77.1 ± 16.06	21.3 ± 4.3
HFHS-C	162.3 ± 7.9	192.6 ± 17.3	216.3.6 ± 16.2	227.6 ± 33.0*	247.3 ± 36.8^**^	85 ± 25.16	21.5 ± 3.7
HFHS	160.5 ± 2.42	198 ± 8.6	220 ± 18.8	281.1 ± 23.2^##^	321.6 ± 27.1^##^	161.1 ± 17.02	20.2 ± 2.3
HFHS-A	163 ± 7.09	193 ± 17.47	210 ± 14.79	232 ± 28.12*	255 ± 30.31^**^	92 ± 21.55	21.5 ± 5.4

Mean ± SD were used to show outcomes in every group (n = 6 wistar rats in each group). *P < 0.05; ^**^P < 0.01; ^***^p<0.001 difference is significant in comparison to group of rats taken HFHS. ^#^ P < 0.05; ^##^P < 0.01; ^###^P < 0.001 difference is significant in comparison to group of rats taken normal fat diet.

**Fig. 1:**
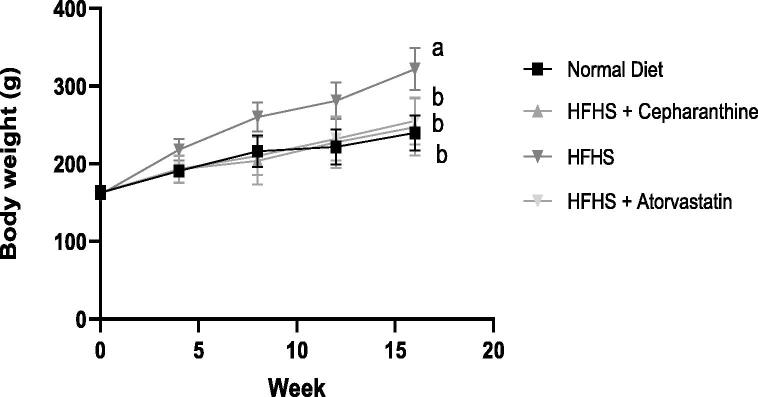
Effect of HFHS-C on rat’s body weight and weight gain.

Compared to the NFD group, a dramatic increase in the HFHS group's weight gain was observed. By our expectations, the caloric intake of rats fed an HFHS was considerably more than that of rats fed in ND (p > 0.05). In contrast, rats given Cepharanthine did not gain weight following a high-fat diet (Fig. 1) regardless of their food intake. Compared to rats kept on the HFHS only, the rats with the HFHS treated with Cepharanthine prevented an increase in body weight gain (p > 0.05). Despite the food available to the animals, no persistent differences were observed in the daily food intake among the four groups, which indicated that Cepharanthine's effects on weight loss were not due to a reduction in energy intake. In the HFHS and HFHS + Cepharanthine groups (Table 4), no substantial variation was observed in the weight of the kidney, indicating that Cepharanthine does not produce any harmful effects. On the other hand, our study observed a Gonadal fat mass index reduction in the HFHS group (Table 4).

### 3.2 In rats fed HFHS, Cepharanthine prevented hyperlipidaemia symptoms

Serum lipid concentrations were measured at week 0 and 16 of the experimental period to investigate Cepharanthine's effect on serum lipid levels (Table 2, Table 3). All four groups of rats had similar initial blood lipid profiles (p > 0.05). In contrast, the rats fed an HFHS diet showed dramatically higher serum TG levels after 16 weeks than rats fed an ND, indicating that the HFHS group was developing hyperlipidemia symptoms (p < 0.05). It is noteworthy that Cepharanthine significantly prevented the rise in serum LDL-C, TC, and TG levels compared with the HFHS group (p < 0.05). Nevertheless, Cepharanthine did not significantly affect HDL-C serum levels compared to the HFHS group in our study.

**Table 2:** Baseline levels of AST, ALT, HDL, LDL-C, TC, TG of rats (n = 6) at the start of the experiment.

Group	TG (mg/dl)	TC (mg/dl)	HDL-C‘ (mg/dl)	LDL-C (mg/dl)	ALT (U/L)	AST (U/L)	Atherosclerosis index	
							TC/HDL-C	LDL/HDL-C
NFD	80.6 ± 5.4	37.3 ± 3.9	14.5 ± 1.8	8 ± 1.4	22.5 ± 2.0	32.8 ± 3.5	2.57 ± 2.85	0.55 ± 1.6
HFHS-C	83 ± 12.5	35.1 ± 4.2	18 ± 1.6	9.6 ± 1.3	23.8 ± 3.6	36.8 ± 2.7	1.95 ± 2.9	0.53 ± 1.45
HFHS	84.8 ± 7.9	42.1 ± 3.86	19.5 ± 1.0	10 ± 1.4	25.1 ± 1.7	35.8 ± 3.0	2.15 ± 2.4	0.51 ± 1.55
HFHS-A	82 ± 11.25	34 ± 4	18 ± 1.89	10 ± 1.21	24 ± 3.02	37 ± 3.24	1.88 ± 2.94	0.55 ± 1.55

Mean ± SD were used to show outcomes in every group (n = 6 wistar rats in each group). *P < 0.05; ^**^P < 0.01; ^***^p<0.001 difference is significant in comparison to group of rats taken HFHS. ^#^ P < 0.05; ^##^P < 0.01; ^###^P < 0.001 difference is significant in comparison to group of rats taken normal fat diet.

**Table 3:** Effects by high-fat diet on AST, ALT, HDL, LDL-C, TC, TG of rats (n = 6) at the end of the experiment (16th week).

Group	TG (mg/dl)	TC (mg/dl)	HDL-C‘ (mg/dl)	LDL-C (mg/dl)	ALT (U/L)	AST (U/L)	Atherosclerosis index	
							TC/HDL-C	LDL/HDL-C
NFD	90 ± 5.5^***^	65.5 ± 7.0^***^	22.1 ± 1.6	27.5 ± 2.7^***^	134.1 ± 4.1^***^	168 ± 3.6	2.96 ± 4.3	1.24 ± 0.8
HFHS-C	109.3 ± 14.2^#***^	70.1 ± 6.7^**^	24.1 ± 0.7^#^	24.1 ± 2.1^***^	123.6 ± 4.5^***^	172.3 ± 10.2	2.90 ± 3.7	1 ± 1.4
HFHS	192.1 ± 12.8^###^	85.3 ± 6.8^###^	24 ± 1.2	41.8 ± 4.7^###^	162.6 ± 10.1^###^	166.8 ± 8.2	3.55 ± 4.0	1.74 ± 2.95
HFHS-A	105 ± 10.73^***^	66 ± 5.74^**^	25 ± 0.95^##^	24 ± 2.79^***^	128 ± 5.70^***^	179 ± 6.99	2.64 ± 3.34	0.96 ± 1.82

Mean ± SD were used to show outcomes in every group (n = 6 wistar rats in each group). *P < 0.05; ^**^P < 0.01; ^***^p<0.001 difference is significant in comparison to group of rats taken HFHS. ^#^ P < 0.05; ^##^P < 0.01; ^###^P < 0.001 difference is significant in comparison to group of rats taken normal fat diet.

**Table 4:** Effect by HFHS-C on the weight of kidney and liver, Gonadal Fat mass, Hepatic Cholesterol, and Hepatic Triglycerides at the end of the experiment (n = 6).

Group	Liver weight (%)	Kidney weight (%)	Hepatic triglycerides (mg/dl)	Hepatic cholesterol (mg/dl)	Gonadal Fat mass	
					Total mass (g)	Fractional mass (g/100 g)
NFD	3.0 ± 0.16^**^	0.64 ± 0.01	928.6 ± 92.8^***^	12.0 ± 1.78^***^	10.33 ± 1.8^***^	4.0 ± 0.46^***^
HFHS-C	3.9 ± 0.16^#*^	0.62 ± 0.01	1423.8 ± 63.0^###***^	72.3 ± 4.2^###***^	5.51 ± 0.87^###*^	2.58 ± 0.44^###**^
HFHS	5.0 ± 0.41^##^	0.62 ± 0.01	2123.0 ± 211.8^###^	50.0 ± 4.2^###^	3.63 ± 0.54^###^	1.5 ± 0.41^###^
HFHS-A	4.9 ± 0.47^##^	0.61 ± 0.02	1419 ± 62.66^###***^	70 ± 4.02^###***^	5.3 ± 0.83^###^	2.4 ± 0.47^###**^

Mean ± SD were used to show outcomes in every group (n = 6 wistar rats in each group). *P < 0.05; ^**^P < 0.01; ^***^p<0.001 difference is significant in comparison to group of rats taken HFHS. ^#^ P < 0.05; ^##^P < 0.01; ^###^P < 0.001 difference is significant in comparison to group of rats taken normal fat diet.

### 3.3 Effect on SOD and MDA antioxidant activity

**Fig. 2:**
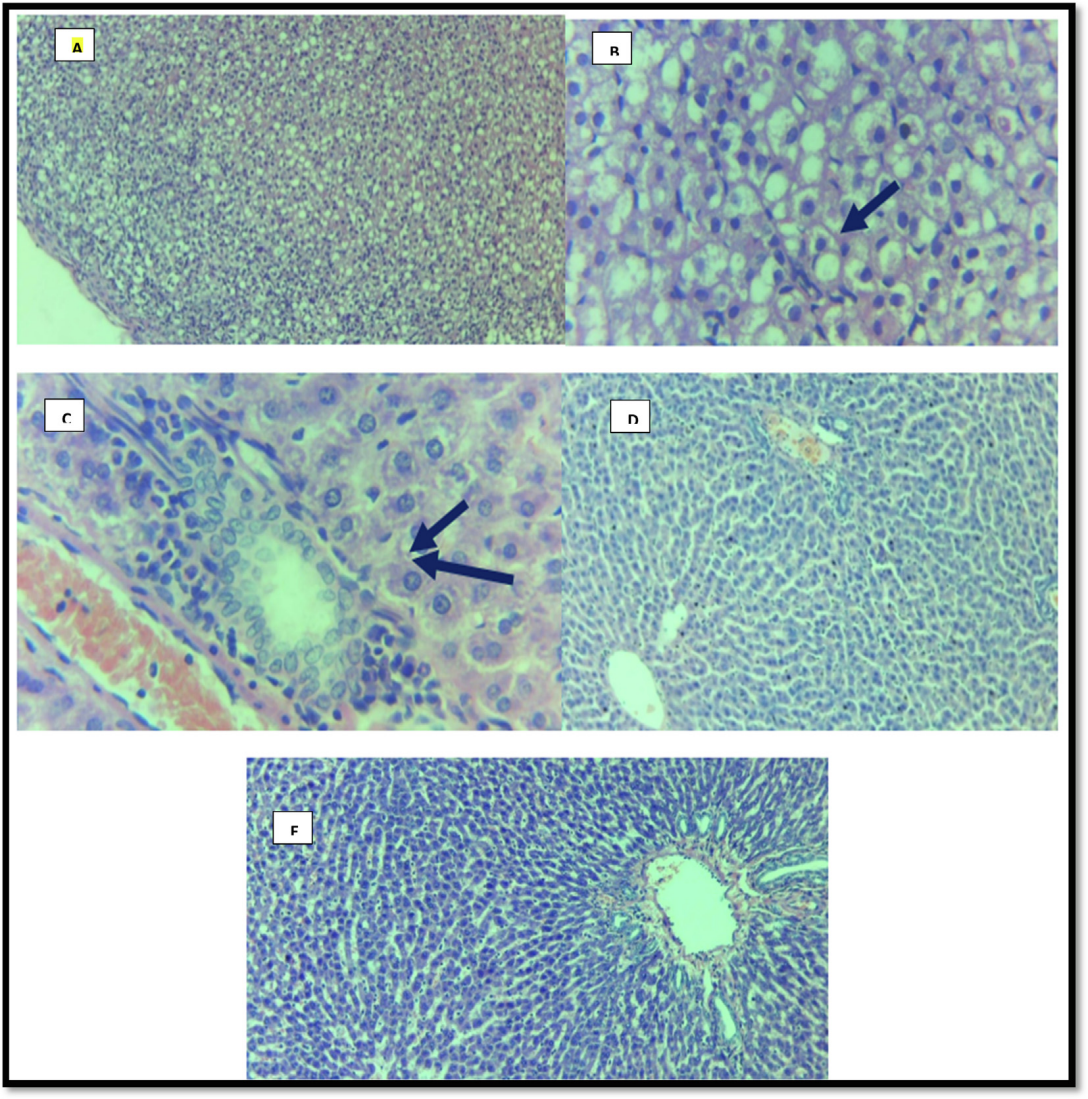
Effect of Cepharanthine on liver antioxidant activity of rats. (n = 6 wistar rats in each group). *P < 0.05; ^**^P < 0.01; ^***^p < 0.001 difference is significant in comparison to group of rats taken HFHS. ^#^ P < 0.05; ^##^P < 0.01; ^###^P < 0.001 difference is significant in comparison to group of rats taken normal fat diet.

The MDA content in the group of rats that have taken a diet rich in high fat and high sugar is significantly increased (P < 0.001), whereas the SOD antioxidant activity is significantly decreased compared to the normal fat diet group. The MDA content in rats treated with cepharanthine declined significantly (P < 0.001) than the HFHS group, whereas SOD activity was significantly increased (Fig. 2).In our rats, these results confirm cepharanthine antioxidant properties.

### 3.4 Cepharanthine as a diabetes prevention agent

Cepharanthine-treated rats showed improved levels of glucose than the HFHS group. Fasting serum insulin and blood glucose levels were used to calculate insulin resistance. HFHS group animals were observed to have high insulin resistance, which was partially alleviated following treatment with cepharanthine. (Table 6).

### 3.5 Effect on the gene expression of ABCC10

**Fig. 3:**
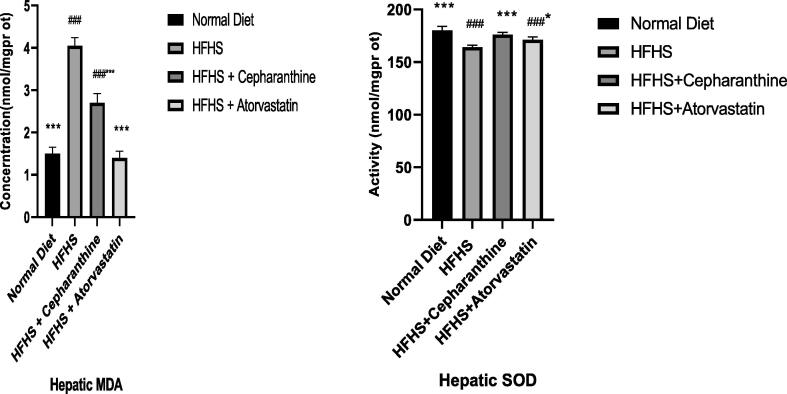
Effect of HFHS-C on the gene expression of ABCC10. (n = 6 wistar rats in each group). *P < 0.05; ^**^P < 0.01; ^***^p < 0.001 difference is significant in comparison to group of rats taken HFHS. ^#^ P < 0.05; ^##^P < 0.01; ^###^P < 0.001 difference is significant in comparison to group of rats taken normal fat diet.

The hepatic gene expression of ABCC10 was significantly increased in animals fed a high-fat sucrose diet by 2.5 times (p < 0.01) vs those who took a normal fat diet. In the rats who used cepharanthine and a high-fat high sucrose diet, gene expression of ABCC10 was significantly increased by 1.6 times versus the NFD rats but significantly down-regulated by 22 % compared to the high-fat high sucrose diet rats (Fig. 3).

### 3.6 Relation between hepatic ABCC10 gene and lipid profile and hepatic enzymes in groups

The expression of ABCC10 gene correlated significantly with serum TC, serum TG levels (r = 0.453) (p = 0.026) (r = 0.615) (p < 0.001). In addition, liver enzymes (ALT) were significantly correlated with ABCC10 expression (r = 0.452) (p = 0.027) (Table 5).

**Table 5:** Correlation between Hepatic ABCC10 and Lipid profile and liver enzymes.

	Hepatic ABCC10
TC	0.453*
HDL	0.558**
LDL	0.417*
TGs ALT AST	0.615** 0.452* 0.202

Pearson correlation (r),

* P < 0.05.

** P < 0.01.

**Table 6:** Effect of HFHS-C on glucose, insulin and HOMA-IR.

Group	Fasting Blood glucose (mg/dl)	Fasting Blood Insulin (ng/ml)	HOMA-IR				
NFD	80 ± 1.87^***^	0.4 ± 0.12^***^	0.09 ± 0.02^***^				
HFHS-C	102 ± 3.65^***###^	0.6 ± 0.05^##***^	0.17 ± 0.02^##***^				
HFHS	130 ± 3.38^###^	1.4 ± 0.12^###^	0.46 ± 0.05^###^				

Mean ± SD were used to show outcomes in every group (n = 6 wistar rats in each group). *P < 0.05; ^**^P < 0.01; ^***^p<0.001 difference is significant in comparison to group of rats taken HFHS. ^#^ P < 0.05; ^##^P < 0.01; ^###^P < 0.001 difference is significant in comparison to group of rats taken normal fat diet.

### 3.7 Ceparanthine prevention in making liver fatty followed by high fat and high sugar diet

**Fig. 4:**
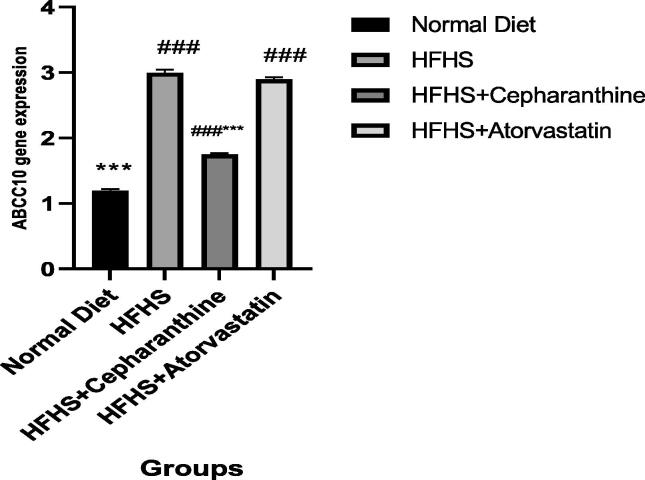
Effect of Cepharanthine on Liver Histopathology.

It was found that a high-fat diet elevated the hyperlipidemia symptoms and body weight and induced the development of fatty liver steatosis. According to Fig. 4, compared to rats on NFD, the rats taking HFHS established fatty liver, distinguished by the increased size of the liver, hepatic swelling with increased serum ALT levels, and TG levels. In addition, the cepharanthine-treated rats showed lower liver TG levels and weight indexes than the HFHS group, although they were still higher than that in the NFD group. On the other hand, among the HFHS and Cepharanthine groups, serum AST levels were considerably lower in the HFHS group (Table 2). Similarly, the HFHS group also displayed lower hepatic cholesterol levels than the cepharanthine group. Remarkably, the livers of the rats on HFHS with Cepharanthine showed normal morphology, with most cells showing a similar size and appearance to the NFD group (Fig. 4).

Fig. 4 shows Light microscopic histopathological photographs of the liver from male adult rats (HE × 40). Section of liver tissue **(A)** and **(B)** showing fatty change, i.e., steatosis (arrow). Section of liver tissue **(C)** showing chronic inflammation (arrow). Section of liver tissue **(D and E)** showing liver parenchyma with normal architecture. **A, B, C;** HFHS Group; **D**; HFHS-C Group, **E**; NFD Group.

## 4 Discussion

The current study suggests that consuming HFHS for a long period may increase blood lipid levels. For our research and practical experiments, we established a model of hyperlipidemic rats by giving the animals HFHS to see and observe the effects of Cepharanthine as a therapeutic agent on hyperlipidemia. Our study confirms that Cepharanthine effectively reduced LDL-C levels, cholesterol levels, and serum TG in hyperlipidemic rats. The triglyceride level was also decreased in the livers of the cepharanthine group compared to the HFHS group. The ultrastructure of liver tissue was examined after the treatment with cepharanthine, and it was noted that lipid droplets significantly decreased after the treatment. Accordingly, it has been demonstrated through these results that Cepharanthine exerts hypolipidemic effects, which could, later on, convey further safety benefits in the prevention of atherosclerosis, which is similar to the mechanism of atorvastatin, the drug that has shown serious improvements in the prevention of heart disease. Also, Cepharanthine showed anti-obesity effects after 16 weeks of oral treatment, as the gain of weight in the cepharanthine group was significantly reduced than that of the HFHS group.

Our research shows that long-term elevated blood lipid levels are implicated in developing liver fibrosis while they can also cause liver damage and fatty liver. It is also known that mitochondria are the organelles responsible for producing ATP, which is essential to all forms of life. Additionally, the metabolism of cell energy heavily depends on the beta-oxidation process of fatty acids. In several publications, the ultrastructure of mitochondria appears to be altered in hyperlipidemic patients. A dysfunctional mitochondrial system can produce excessive ROS and disrupt fatty liver homeostasis, resulting in lipid peroxidation, cytokine release, and cell death(Begriche et al., 2006). Animals in the high-fat diet group had reduced mitochondria and endoplasmic reticulum, significant liver damage, and elevated lipid droplets. Cepharanthine supplementation reduced fatty liver, significantly reduced lipid accumulation, prevented the inflammation of the liver, and caused improvement in levels of ER and mitochondria in the cell. Also, the concentration of triglycerides in liver tissue of the cepharanthine treated group was significantly lower than that of the group who consumed high fat/high sugar diets. Cepharanthine appears to play a role in regulating the levels of lipids. In addition, Cepharanthine may also act as a regulator of other lipids in the plasma, such as triglycerides and phospholipids. These lipids are known atherogenic particles, so their regulation may prove crucial in treating cardiovascular diseases such as atherosclerosis(Zilversmit, 1995).

Additionally, different levels of blood lipids are considered the chief risk factor for diseases associated with lipid metabolism; elevated levels of LDL-C, serum TC, and TG, as well as lower levels of HDL-C, are some of the major signs and symptoms of hyperlipidemia (Manting et al., 2011). This study confirmed previous studies that HFHS causes elevated serum LDL-C, serum cholesterol, and serum TG levels in the HFHS groups (Méndez, Pazos, Molinar-Toribio, Sánchez-Martos, Gallardo, Nogués, et al., 2014). Also, for atherosclerotic cardiovascular disease, risk indicators are LDL-C, cholesterol, and serum TG levels(Deedwania et al., 2016). However, reducing the TG and LDL-C levels can help improve the risk of acute coronary syndromes and reduce vascular disease(Wada et al., 2020). Our study found that the LDL-C, blood cholesterol, and serum TG levels were lower in the cepharanthine group than in the high-fat diet group. Nevertheless, HDL-C levels did not differ significantly from those in the atorvastatin group.

Furthermore, cepharanthine has been observed to prevent the gain of weight in animals who consumed high sugar diet. This is similar to an earlier study, which showed that consumption of high-fat high sucrose increased the body's weight (Méndez et al., 2014). The high-fat diet group gained more weight than the other groups. The cepharanthine group displayed much lower weight gain than the HFHS group and demonstrated specific effects in reducing the overall weight gain. On the contrary, it did not appear to affect food intake substantially. In our study, cepharanthine was found to have anti-obesity effects, which could be explained by the fact that cepharanthine promotes the utilization and breakdown of excess body fat.

Increasing levels of oxysterols are associated with high cholesterol and triglycerides levels in rats fed with a high-fat, high sucrose diet. Oxysterols interfere with ABC transporters. ABCA1 and ABCG1 affect cholesterol efflux, whereas ABCG5 and ABCG8 influence hepatic cholesterol excretion and absorption(Li et al., 2017).

In the present study, we found significant changes in the gene expression of hepatic ABCC10 in rats fed a high-fat high sucrose diet or those fed a high-fat high sucrose diet and treated with cepharanthine compared to normal controls. Cepharanthine inhibits ABCC10 effectively (Zhou et al., 2009). We found that cepharanthine has an effective role in maintaining cell cholesterol and lipid homeostasis by promoting reverse cholesterol transport. This is shown in the present study when TC, TGs, LDL-C, and ALT levels are significantly lower in the HFHS + cepharanthine group than in the HFHS group. The results confirm ABCC10 role in the prevention of dyslipidemia and obesity.

Rats fed a high fat, high sucrose diet had higher liver enzyme levels than rats fed a low fat, low sucrose diet. Earlier studies have shown a strong association between elevated liver enzymes and lipid profile(Kathak et al., 2022). Furthermore, a significant correlation was found between serum ALT levels and gene expression of hepatic ABCC10, suggesting that overexpression of these drug transporters is associated with liver damage. This study confirms previous findings(Borel et al., 2011).

These results indicate that endogenous oxysterols or high fat, high sucrose diet are responsible for the up-regulation of ABCC10 gene expression. Additionally, ABCC10 mediates multidrug resistance in cancer cells; dyslipidemia may contribute to multidrug resistance in cancer cells via ABCC10 up-regulation.

The data obtained from this study will be invaluable for studying molecules or inhibitors that may be used to treat these diseases. Additionally, many patients fail to reach current cholesterol, triglycerides, and LDL target levels due to intolerance to conventional statin therapy or an insufficient response to it (Boekholdt et al., 2014). In light of this, it is necessary to devise new approaches and methods to treat hyperlipidemia. Cepharanthine is one potentially promising candidate in this regard for managing patients suffering from these conditions and improving their symptoms.

## 5 Conclusion

It has been demonstrated that cepharanthine reduces serum lipid profiles and prevents weight gain in the liver by preventing hepatic fatty deposition. Cepharanthine is a potential drug candidate to treat or prevent obesity and hyperlipidemia. Further research is required to examine the effects of cepharanthine on obesity, antioxidant activity, and hyperlipidemia.

## Declaration of Competing Interest

The authors declare that they have no known competing financial interests or personal relationships that could have appeared to influence the work reported in this paper.
